# Use of *in vitro* methodology to investigate phthalate effects on the differentiation of seminiferous tubule-associated stem cells to form Leydig cells and on the Leydig cells derived from the stem cells

**DOI:** 10.3389/ftox.2024.1352294

**Published:** 2024-02-01

**Authors:** Kassim Traore, Barry Zirkin

**Affiliations:** ^1^ Jerry M. Wallace School of Osteopathic Medicine, Campbell University, Lillington, NC, United States; ^2^ Duquesne University College of Osteopathic Medicine, Pittsburgh, PA, United States; ^3^ Johns Hopkins Bloomberg School of Public Health, Baltimore, MD, United States

**Keywords:** phthalates, Leydig cells, stem cells, tubule culture, steroidogenesis

## Abstract

**Introduction:** Leydig cells isolated from the testis are able to sustain high levels of testosterone production *in vitro*, but only for up to 3 days. Such cells are valuable for addressing the acute effects of chemicals on steroidogenic function, but not for repeated or chronic effects. Methodology is now available by which adult Leydig cells can be derived *in vitro* from seminiferous tubule-associated stem cells. In contrast to isolated Leydig cells, the Leydig cells derived in this way can synthesize and secrete high levels of testosterone for months. Herein, we asked whether this system might be used to address the effect of mono-(2-ethylhexyl) phthalate (MEHP) exposure on the formation of Leydig cells from tubule-associated stem cells, and on the Leydig cells after their formation.

**Methods:** Adult Brown Norway rats received an intraperitoneal injection of ethane dimethanesulfonate (EDS) to eliminate the existing Leydig cells. Seminiferous tubules then were isolated and cultured in medium containing Insulin-Transferrin- Selenium (ITS), Smoothened Agonist (SAG), and luteinizing hormone (LH).

**Results:** Culture of the tubules for 8 weeks resulted in the formation of cells on the surfaces of the tubules that stained for CYP11A1 and STAR and produced high levels of testosterone. When the tubules were cultured in medium containing increasing concentrations of MEHP, concentration-dependent effects on Leydig cell formation occurred. To determine the effect of MEHP on newly produced Leydig cells, tubules were cultured for 8 weeks in the absence of MEHP, resulting in the formation of adult Leydig cells, and then in medium containing increasing concentrations of MEHP. Concentration-dependent decreases in testosterone production by the adult Leydig cells were seen, and these decreases proved to be reversible.

**Discussion:** The use of this new system should make it possible to determine the mechanisms by which acute, repeated, or chronic exposures to increasing concentrations of MEHP and/or exposure to other chemicals affect the formation of Leydig cells from stem cells, as well as the steroidogenic function of adult Leydig cells.

## Introduction

Testosterone, produced by the testicular Leydig cells, is essential for the development of the male reproductive system and for the maintenance of important reproductive and quality of life functions (O'Donnell et al., 2017; Gurung et al., 2023; Wijesinha et al., 2013; Nieschlag and Nieschlag, 2019; Papadopoulos and Zirkin, 2021). Much of what is known about Leydig cell steroidogenic function has come from *in vivo* studies, *in vitro* studies of cell lines, and primary Leydig cells. *In vivo* studies can be quite informative, but such studies typically cannot specifically address the direct effects of exogenous substances on the cells of interest. Long-term studies have been conducted in animals or by using transformed cell lines as alternatives to primary cells (Ascoli, 1981). An advantage of cell lines is that they typically are able to produce steroids *in vitro* for long periods of time, making it possible to address the effects of acute, repeated, and chronic exposures. However, many among these cells, including the frequently utilized MA-10 cells, do not produce testosterone as their final product (Ascoli, 1981; Midzak et al., 2011). Moreover, whereas primary Leydig cells rarely turn over, the transformed cells typically turn over with frequency, thus potentially creating difficulty in interpreting results.

Primary Leydig cells can be isolated directly from the testes. In response to luteinizing hormone (LH), these cells can produce testosterone at levels comparable to testosterone production *in vivo*. However, high levels of testosterone are produced only for as long as up to about 3 days.

(Klinefelter and Ewing, 1988; Carney et al., 2014). This is not long enough for studies that seek to address such issues as the effects of chronic or repeated exposures to environmental toxicants on Leydig cell steroidogenic function. It has been clear for decades that the availability of LH-responsive Leydig cells with the ability to produce high, stable levels of testosterone for long periods of times *in vitro* might be particularly valuable for assessing such effects and the mechanisms involved.

Phthalates are industrial plasticizers commonly used to increase the flexibility of polyvinyl chloride (PVC) products, and as additives to consumer products including cosmetics, dietary supplements, and medications (Kohn et al., 2000). Present in human amniotic fluid, placenta, urine, blood, saliva and other bodily fluids (Silva et al., 2004; Adibi et al., 2009; Jeong et al., 2011; Yang et al., 2015), phthalates have been shown to exhibit antiandrogenic properties in humans (Borch et al., 2006; Radke et al., 2018; Radke et al., 2019; Willson, 2021) and to cause developmental and reproductive toxicity in rodents (Repouskou et al., 2021; Willson, 2021). As yet, however, it has been difficult to conduct long-term mechanistic studies of phthalate effects on the development of Leydig cells and on their adult steroidogenic function.

In the present study, we addressed the effects of chronic exposure to the phthalate mono-2-ethylhexyl phthalate (MEHP), the active metabolite of di-2-ethylhexyl phthalate (DEHP), on the formation of Leydig cells and on adult Leydig cell function using *in vitro* methods by which testosterone-producing Leydig cells are produced from stem cells associated with isolated seminiferous tubules (Odeh et al., 2014; Li et al., 2016; Chen et al., 2017). In contrast to Leydig cells isolated directly from testes, the tubule-associated Leydig cells produce and sustain high levels of testosterone production in response to LH *in vitro* for many months. Once formed, the Leydig cells derived from the stem cells *in vitro* rarely turn over, which is in contrast to MA-10 cells, and testosterone production per cell in response to LH is comparable to its production by isolated primary Leydig cells for many months rather than days (Zirkin and Papadopoulos, 2018). We used this system to address the effects of MEHP on the formation of adult Leydig cells from stem cells on the surfaces of the seminiferous tubules, and on testosterone production by the adult Leydig cells derived from the tubule-associated stem cells. Additionally, the ability to conduct long-term studies also made it possible to address the possible reversibility of MEHP effects.

## Materials and methods

### Animal care

Brown Norway rats were purchased from Charles River (Kingston, MA), housed at 22°C under 12-h light/dark cycles, and fed *ad libitum*. Prior to collection of blood and tissues, the rats were euthanized by decapitation. Animals were handled according to protocols approved by the Campbell University and/or Duquesne University Animal Care and Use Committees.

### MEHP effects on Leydig cell formation from seminiferous tubule-associated stem cells

Previous studies demonstrated that stem cells are present on the surfaces of seminiferous tubules and that, under appropriate conditions, these cells are able to give rise to adult Leydig cells (Stanley et al., 2012). In the current study, adult (4-month-old) Brown Norway rats received a single intraperitoneal injection of ethane dimethanesulfonate (EDS) at 85 mg/kg body weight to eliminate the existing Leydig cells from the testes (Morris et al., 1986; Kelce et al., 1991). Seminiferous tubules then were isolated and cultured in medium containing Insulin-Transferrin-Selenium (ITS, 10 μg/mL), Smoothened Agonist (SAG, 0.5 μM), and luteinizing hormone (LH, 100 ng/mL). The formation of steroid-producing-cells by the tubules was determined by the appearance of cells that express CYP11A1 and STAR, and by testosterone production. The expressions of CYP11A1 and STAR were examined by immunochemical methods, using rabbit anti-rat CYP11A1 or STAR antibodies (Cell Signaling Technology, MA). Tubules were suspended in permeabilization buffer containing rabbit anti-CYP11A1 or STAR antibodies at a dilution of 1:1,000 and incubated for 60 min. Subsequently, the samples were incubated in goat anti-rabbit IgG linked to alkaline phosphatase (Invitrogen, Life Science Technology, MA). For detection of CYP11A1 or STAR, tubules were washed 3 times and then exposed to the alkaline phosphate substrate BCIP/NBT. To measure testosterone production, seminiferous tubules were cultured in differentiation medium containing luteinizing hormone (LH, 100 ng/mL). Testosterone in the culture medium was assessed by enzyme-linked immunosorbent assay (ELISA) using the Biomatik Rat Testosterone kit (Ontario, Canada). Using these methods, the effect of MEHP (1 or 5 μM) exposure on the formation of steroid-producing Leydig cells was determined.

### MEHP effects on Leydig cells derived from seminiferous tubule-associated stem cells

Seminiferous tubules were cultured in differentiation medium for 2 months, resulting in the production of Leydig cells from the tubule-associated stem cells. To determine MEHP effects on the formed Leydig cells, the tubules, with their associated Leydig cells, then were cultured in differentiation medium containing MEHP (0, 5 or 100 µM) for 24, 48 or 72 h. Using techniques described above, CYP11A1 and STAR expressions were examined, and testosterone concentration in the medium was determined.

### Statistical analysis

Data are expressed as the mean ± standard error of the mean (SEM). Group means were evaluated by one-way ANOVA. Comparisons between groups were made using an independent *t*-test. The null hypothesis of no effect was rejected at *p* < 0.05 as previously described (Zhou et al., 2019).

## Results

### Leydig cell formation and steroidogenic function

**FIGURE 1:**
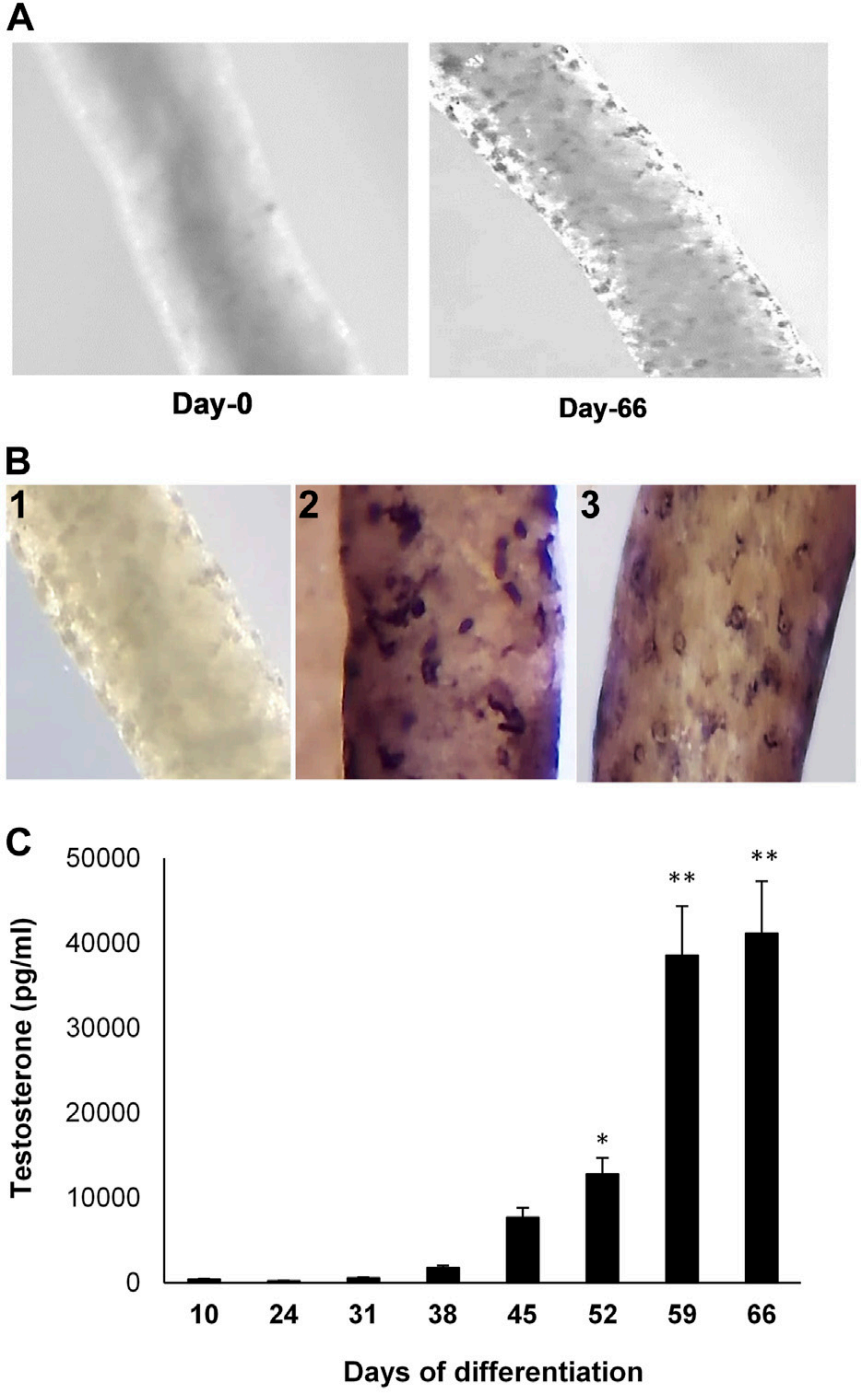
Leydig cell formation on the surface of seminiferous tubules. **(A)** Seminiferous tubules, isolated from EDS-treated young Brown Norway rat testes, were cultured in LH-containing differentiation medium for 0 (Day-0) to 66 (Day-66) days. **(B)** 1) Seminiferous tubule stained with anti-rabbit IgG (isotype-control). 2) Tubule cultured in LH-containing medium for 66 days and then stained for CYP11A1. 3) Tubule cultured in LH-containing medium for 66 days and then stained for STAR. **(C)** Testosterone production, over time, by cells formed on the surfaces of tubules that were cultured in LH-containing differentiation medium through Day 66. Asterisks indicate significant difference from control.

**FIGURE 2:**
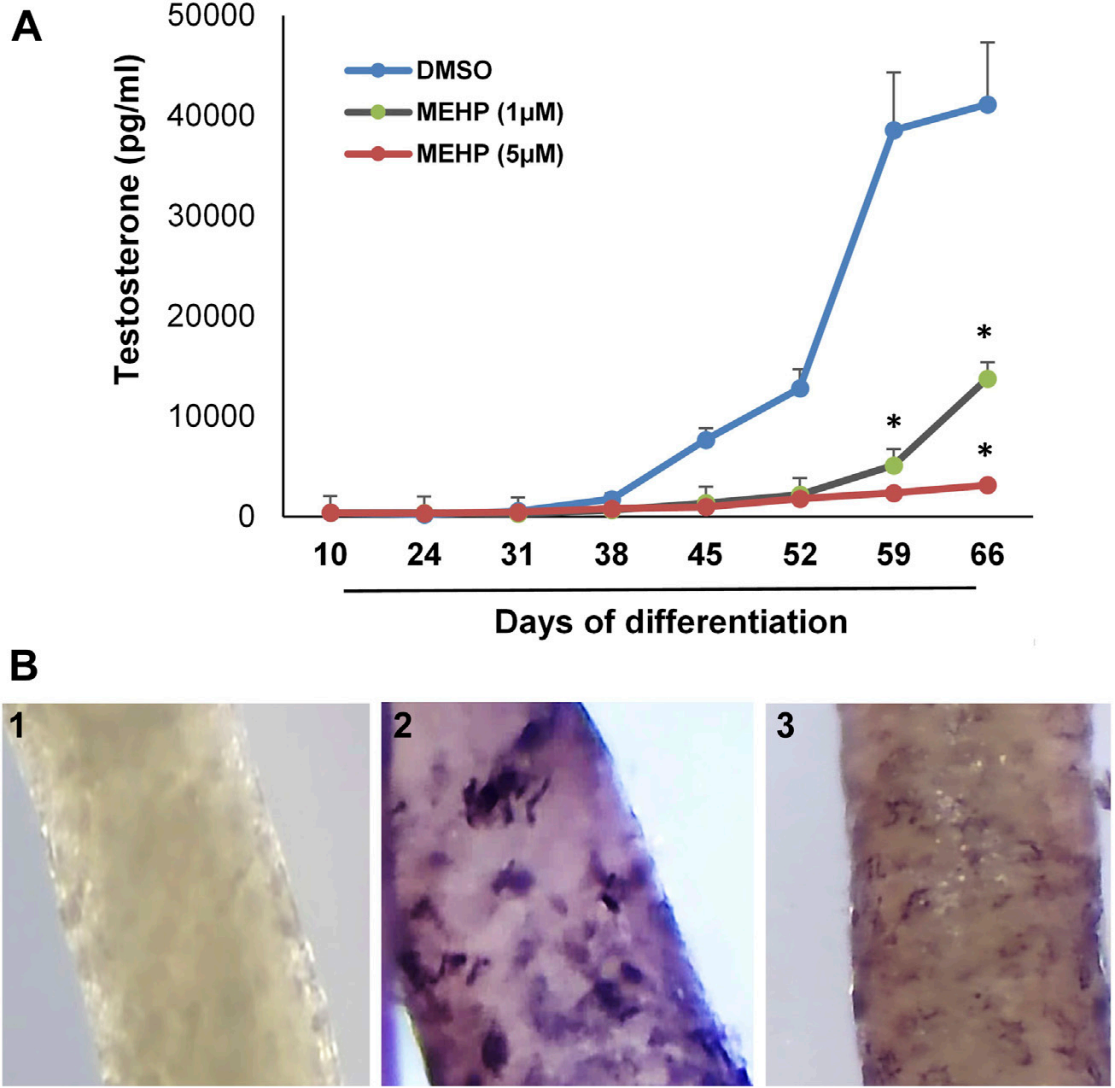
Effect of MEHP on Leydig cell formation and steroidogenic function. Seminiferous tubules isolated from EDS-treated rats were cultured in medium containing DMSO (control) or DMSO plus increasing concentrations of MEHP (1 or 5 µM). **(A)** Testosterone production over time by cells associated with tubules cultured in LH-containing differentiation medium through Day 66. Asterisks indicate significant reductions compared to DMSO controls. **(B)** 1) Tubule cultured for 66 days and stained with anti-rabbit IgG (isotype-control). 2) Tubule cultured in LH-containing medium for 66 days without MEHP and then stained for CYP11A1. 3) Tubule cultured with medium containing MEHP for 66 days and then stained for CYP11A1.

**FIGURE 3:**
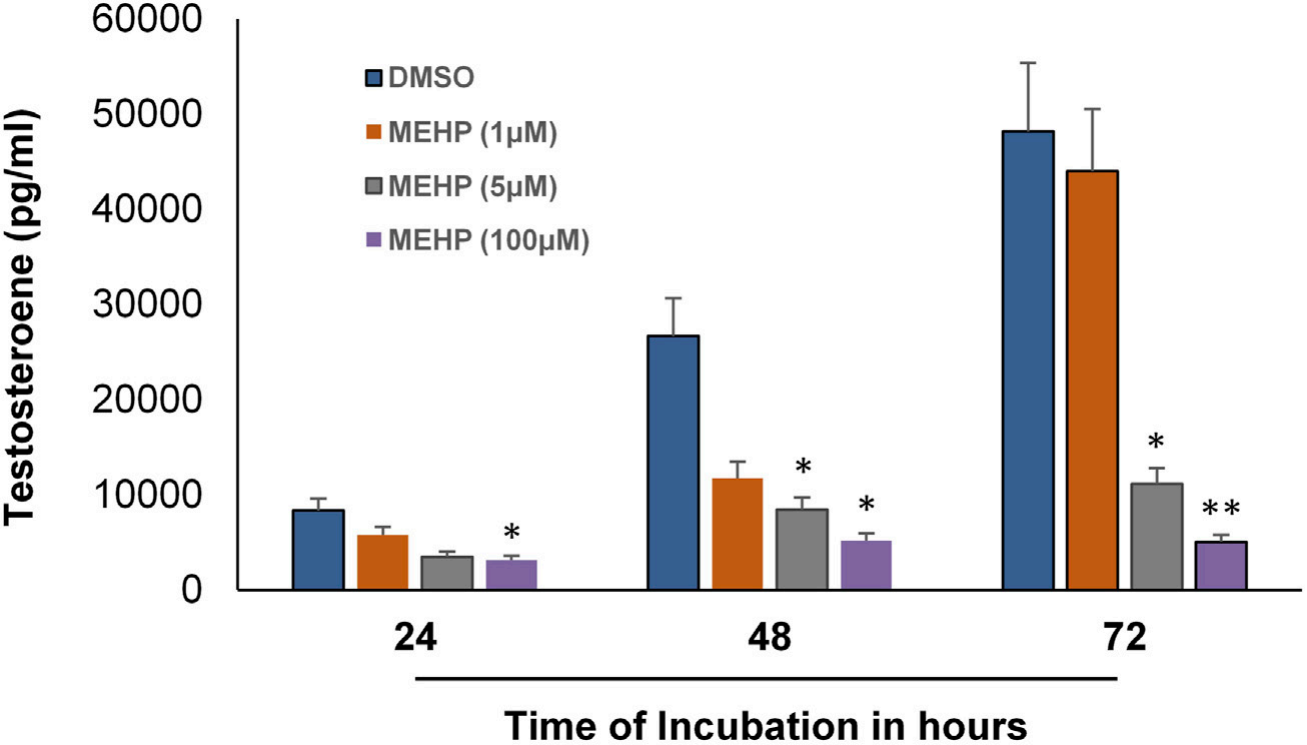
Effect of MEHP on adult Leydig cell steroidogenic function. Seminiferous tubules isolated from EDS-treated rats were cultured in differentiation medium for 2 months. The tubules, with Leydig cells having formed on their surfaces, were then cultured in fresh medium in the absence (DMSO, Control) or presence of MEHP (1, 5, or 100 µM) for 24, 48 or 72 h. Testosterone was measured in the medium. Asterisks indicate significant difference from controls.

Seminiferous tubules were isolated from EDS-treated Brown Norway rat testes (Figure 1A, Day 0). Culturing the tubules in LH-containing differentiation medium resulted in the appearance of cells on the tubule surfaces (Figure 1A, Day 66). Whereas the newly isolated tubules did not show presence of cells (Figure 1A), culturing the tubules in LH-containing differentiation medium resulted in cells on the tubule surfaces (Figure 1B-1) that stained for CYP11A1 (Figure 1B-2) and STAR (Figure 1B-3). As seen in Figure 1C, culturing the tubules in LH-containing culture medium resulted in the increasing production of testosterone over time, with significant increases at 52 days of culture and thereafter.

### MEHP effects on stem Leydig cell differentiation and Leydig cell formation

We used the *in vitro* system to assess the effects of MEHP on the formation of Leydig cells from stem cells. Tubules were isolated from EDS-treated rats and cultured for 66 days in medium containing DMSO alone or DMSO plus 1 or 5 μM MEHP. Testosterone production was measured in the medium over time. The presence of MEHP during the differentiation process resulted in MEHP concentration-dependent decreased testosterone production, with significant decreases at both 1 and 5 μM concentrations (Figure 2A). Consistent with reduced testosterone production, decreased immunohistochemical staining for CYP11A, the enzyme that converts cholesterol to pregnenolone within the mitochondria (Zirkin and Papadopoulos, 2018; Papadopoulos and Zirkin, 2021), occurred in response to MEHP (compare control cells, Figure 2B-2) with cells cultured with MEHP; Figure 2B-3. Cell viability was not affected by MEHP exposure (not shown).

### MEHP effects on adult Leydig cells formed from tubule-associated stem Leydig cells

We next sought to determine whether the *in vitro* system could be used to examine the effects of MEHP on testosterone production by the Leydig cells produced from the differentiation of the tubule-associated stem cells. To this end, seminiferous tubules were isolated from EDS-treated rats and cultured in LH-containing differentiation medium for 66 days. The tubules, with Leydig cells having formed on their surfaces, then were cultured in control media (DMSO) or media containing MEHP (1, 5, or 100 μM). Testosterone levels in the culture medium were measured after 24, 48 and 72 h of incubation. As shown in Figure 3, testosterone production increased in the controls with increasing time of incubation through 72 h. When incubated for 48 or 72 h in culture medium containing MEHP, Leydig cell testosterone production was decreased significantly from control levels at 5 and 100 μM MEHP.

### Reversibility of MEHP effects?

**FIGURE 4:**
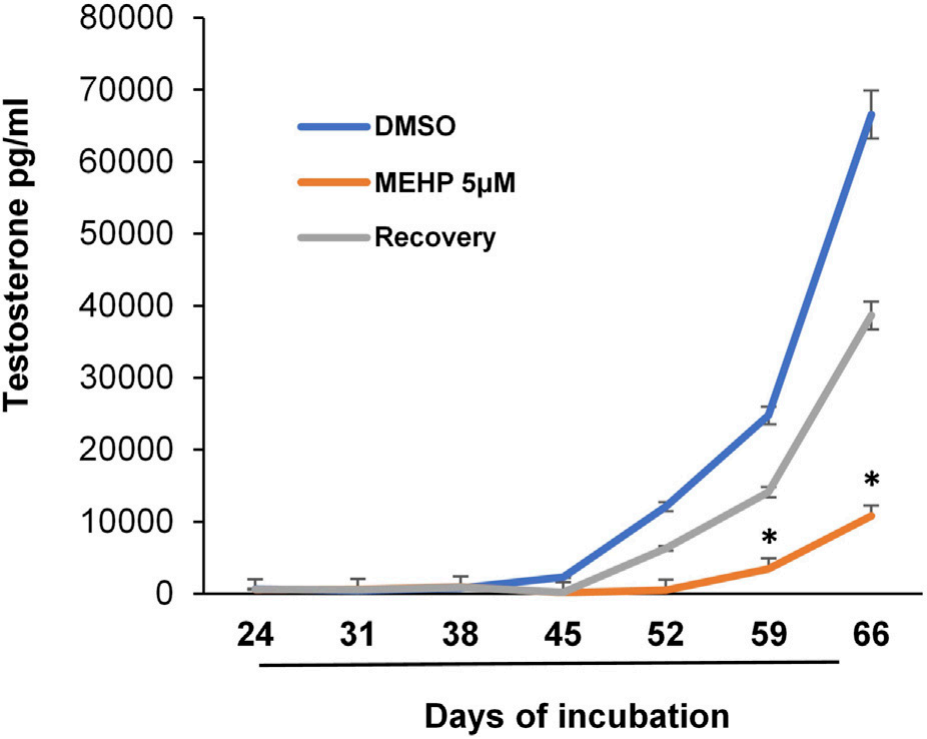
Reversibility of MEHP effect on Leydig cell formation and steroidogenic function. Seminiferous tubules isolated from EDS-treated rats were initially cultured in medium in the absence (DMSO, control) or presence of MEHP (5 µM) for 30 days. At the end of the 30-day period, the tubules were cultured for an additional 36 days in medium that either contained MEHP (MEHP) or did not contain MEHP (Recovery). Testosterone production was measured in the medium over time. Asterisks show significant difference from control.

We recognized that the availability of an *in vitro* system with which Leydig cell function is maintained for relatively long periods time could prove beneficial for addressing not only the effects of chronic or repeated exposures to toxicants and the mechanisms involved, but also possible reversibility. With this in mind, we tested the possible use of the tubule-associated Leydig cell formation system in determining whether the effects of MEHP on differentiating stem Leydig cells might be reversible. Isolated seminiferous tubules were incubated in differentiation medium alone or in medium containing MEHP (5 µM) for 30 days. For the next 36 days, control tubules continued to be cultured in the absence of MEHP, while tubules that had been cultured with MEHP for 30 days were cultured for 36 additional days either in the presence or absence of MEHP. As seen in Figure 4, cells that had been exposed to MEHP for the full 66 days produced significantly less testosterone than the controls. However, testosterone synthesis by the cells that had been exposed to MEHP for 30 days but then were cultured in its absence for the following 36 days was almost comparable to controls, indicating that the effects seen in response to 5 µM MEHP were not permanent.

## Discussion

Our objective was to assess the use of an *in vitro* system involving Leydig cell formation from stem cells on the surfaces of seminiferous tubules (Chen et al., 2017) for determining acute and chronic effects of MEHP on Leydig cell formation and on adult Leydig cell function. After the elimination of Leydig cells from the testes of adult Brown Norway rats with EDS, seminiferous tubule fragments from these rats were cultured in medium containing LH, which resulted in the formation of Leydig cells from tubule-associated stem cells. In one series of experiments, exposure of the tubule fragments to MEHP during the period of stem cell differentiation resulted in concentration-dependent decreases in testosterone production. Decreases might have occurred as a consequence of reduced numbers of stem cells, altered differentiation of the stem cells, or reduced steroidogenesis by the Leydig cells derived from the stem cells. It is now apparent that the use of the *in vitro* system will allow the mechanism by which MEHP affects Leydig cell formation to be determined.

Because the Leydig cells produced by this *in vitro* system sustain high levels of testosterone production for months, its use made it possible to determine both short-term and long-term effects of MEHP on testosterone production by the Leydig cells that resulted from differentiation of the stem Leydig cells. We found that MEHP exposure led to a significant reduction in testosterone production by the Leydig cells, a finding that indicates that MEHP can have a negative impact on mature Leydig cell function as well as on the formation of the adult cells. Additionally, in preliminary studies (results not shown), we found that reduced testosterone production was associated with increased accumulation of MnSOD, suggesting that oxidative stress induced by MEHP might be involved in causing reductions in testosterone. These findings, using the tubule-associated Leydig cells, are consistent with previously reported results of short-term MEHP exposure on Leydig cells isolated from the testis (Wu et al., 2021) and on MA10 Leydig cells (Traore et al., 2021). The ability of the tubule-associated Leydig cells to sustain testosterone production long-term will allow detailed mechanistic studies to be conducted.

The capacity for long-term studies provided by this new system also allowed investigation into the possible reversibility of MEHP effects. We found that after culture of the tubules in MEHP-containing differentiation medium, removal of the MEHP from the medium resulted in significantly increased Leydig cell testosterone production. This result suggests that the detrimental effects of MEHP on stem Leydig cell differentiation and/or on the formed Leydig cells may not be permanent. This reversibility is promising and raises questions about the underlying mechanisms and potential interventions that might mitigate MEHP’s impact on Leydig cells.

## Data Availability

The original contributions presented in the study are included in the article/Supplementary Materials, further inquiries can be directed to the corresponding author.
